# The association of family structure with health behavior, mental health, and perceived academic achievement among adolescents: a 2018 Korean nationally representative survey

**DOI:** 10.1186/s12889-020-08655-z

**Published:** 2020-04-16

**Authors:** Hanul Park, Kang-Sook Lee

**Affiliations:** 1 Department of Preventive Medicine, College of Medicine, The Catholic University of Korea, 222 Banpo-daero, Seocho-gu, Seoul, 06591 South Korea; 2 Department of Public Health, Graduate School, The Catholic University of Korea, 222 Banpo-daero, Seocho-gu, Seoul, 06591 South Korea

**Keywords:** Family structure, Health behavior, Mental health, Perceived academic achievement, Adolescent, Korea

## Abstract

**Background:**

Adolescence is a period during which physical, social, and mental abilities are rapidly developed, and during this time the family environment plays an important role. Differences in health behaviors, mental health, and academic achievement by family structure may affect future families, income, and employment. The purpose of the study was to investigate the association of family structure with health behaviors, mental health, and academic achievement in Korean adolescents.

**Method:**

Data from the 2018 Korean Youth Risk Behavior Web-based Survey were analyzed. The study sample was comprised of 59,096 adolescents. Logistic regression, t-tests, and a variance analysis of a complex sample general linear model were used to examine the association of family structure with health behaviors, mental health, and academic achievement. The significance level was set at *P* < 0.05.

**Results:**

Non-intact families (single-mother families, single-father families, and restructured families) had significantly higher odds of smoking a cigarette, drinking a sip of alcohol, internet use, physical activity, and sexual experience, and mental health issues such as depression, suicidal ideation, perceived stress, and poor perceived health status than intact families (two-parent families). Also, non-intact families were significantly related to low perceived academic achievement compared to intact ones.

**Conclusion:**

This study showed that family structure is a significant factor in adolescent health behavior, mental health, and perceived academic achievement. Adolescents who experience a transition in their family structure may be more vulnerable to health risks and exhibit lower academic achievement than those in an intact family.

## Background

Families are considered as the key to achieving the sustainable development goals [1]. Family interventions in poverty, health, education, gender, and violence problems have proved effective and had a positive impact on education and health. Families are a basic social unit. So, families will inevitably impact the progress or backwardness of communities and societies. Changes in the family structure are an international issue [1]. In Korea, the proportion of single-parent families is increasing for reasons such as divorce, separation, bereavement, and runaways [2]. Statistics Korea reported that the proportion of single-parent families was 9.02% in 2010, 10.5% in 2014, and 10.9% in 2018 among the total families in Korea [3]. Non-intact families are exposed to complex and diverse problems involving poverty [4], employment, and education [5]. In addition, adolescents living with one parent tend to be bullied more often than those living with two parents [6]. Single-parent families would like to make future plans, including social and educational success, for their children, but as long as they face social difficulties, they may not able to plan for the future and must focus solely on their current needs [7]. About 16% of single-parent families in Korea have experienced discrimination from their neighbors, schools, workplaces, public institutions, and relatives, and single-parent families had higher rates of use of child-care institutions for their children than intact families, less frequent leisure activities with their children, and higher rates of children being left alone after school [2].

In 2018, indicators of adolescents’ health behaviors in Korea were reported as 14.9% smoking, 7.9% using of e-cigarettes, 2.9% using heated tobacco products, 42.3% drinking, 1.1% using drugs, 113.4 min per week of internet use, 67.3% having physical activity less than 3 days per week, obesity in 14.4%, and sexual experience in 5.7%. Indicators of mental health of adolescents include depression, 27.1%; suicidal ideation, 13.3%; and above-average stress, 40.4% [8]. The differences in health behavior caused by socioeconomic factors are very complex and are strongly related to mortality and morbidity [9, 10]. Socioeconomic factors, which include family structure, also explain inequality in differences in health behaviors such as tobacco [11], alcohol, and drug use in adolescents [12]. Also, the family structure and parental factors affect adolescents’ physical activity [13], internet addiction [14, 15], weight [16], and well-being [17]. The average monthly income of single-parent families was 219.6 million won, 56.5% of the overall average of 389.0 million won of total household income [2].

Early life is a period during which a person develops physically, socially, and mentally, and during this time the family environment plays an important role [18]. Divorce in a family negatively affects adolescents’ mental well-being and causes stress at the beginning of puberty [19, 20]. Because this is later a risk factor for mental health disorders, adolescents from non-intact families are a particular public health concern [21, 22]. The environment associated with the reconstruction of a new family after a divorce is also important for the well-being of adolescents, and the expectation of adapting to such an environment could have a negative effect on children’s mental health. These effects vary depending on the family structure, which suggests that adolescents from non-intact families are a vulnerable group [23–25]. Also, adolescents with low socioeconomic status (SES) are more frequently exposed to poor psychosocial resources within the family, education, and peer environments [26]. The impact of low SES on psychological characteristics can affect adolescents’ adverse health behaviors such as smoking, drinking, bad diet, and physical inactivity [27–29]. Therefore, it is important to identify vulnerable adolescents and better understand them to mitigate the negative impact of divorce.

Some studies have shown that a non-intact family structure is also related to poor academic achievement [30, 31]. Previous studies showed that schools with high rates of adolescents from single-parent families have lower academic performance than other schools, and adolescents with a single parent were related to poor math and science achievements in 9 out of 11 countries [32, 33]. Most adolescents with a single parent have experienced the disruption of their family and an additional transition of the family structure. The accumulation of turbulence and lack of support in the family experienced by adolescents are related to academic achievement [34]. However, none of these studies specifically examined the association of the family structure with health behaviors, mental health, and academic performance.

Differences in health behaviors, mental health, and academic achievement by family structure may affect future families, income, and employment. Focusing on the relationship between family structure, health behavior, mental health, and academic performance could help to better understand health inequality [6, 7, 11]. Depending on the family structure, adolescents are associated with some negative consequences and have different degrees of disadvantage. Previous studies [5–7, 11, 12] compared the health behaviors of adolescents in single-parent families and two-parent families. However, there was no specific study of the health behavior, mental health and academic achievement of the adolescents of single-mother families, single-father families, and restructured families. Recognizing and understanding the lifestyles of adolescents across various family structures might be effective in improving the lives of adults [17]. The purpose of this study was to investigate the association of family structure with health behaviors, mental health, and academic achievement in Korean adolescents.

## Methods

### Participants

The present study was based on the Korean Youth Risk Behavior Web-based Survey (KYRBS), which is a nationally representative survey of Korean adolescents’ health status [8]. The purpose of the KYRBS is to calculate statistics on the health behaviors of Korean adolescents and to use the data to plan and evaluate adolescent health promotion projects. Every year since 2005, the Korea Centers for Disease Control and Prevention (KCDC) have used anonymous online surveys to ask adolescents to self-report their health status, including smoking, drinking, obesity, nutrition, physical activity, and mental health [35].

This study used data from the 14th KYRBS (2018), which was collected in April 2018 and consisted of 103 questions and 97 indicators. The questions and indicators are revised each year by a committee of experts from each relevant professional field utilizing domestic and international data. The study examined 62,823 students from 400 middle and 400 high schools, with 60,040 students participating (95.6%) [8].

Complex sampling was used to obtain a representative sample of Korean adolescents. The sampling processes were divided into population stratification, sample distribution, and stratified cluster sampling. In the population stratification stage, local groups and schools were used as the stratification variable to minimize sampling error. In the sample distribution stage, the sample size was 400 middle and 400 high schools. The proportion distribution method was applied to be consistent with the population composition and sample composition ratio by the stratification variable. In the stratified cluster sampling, the school was the first unit extracted, followed by the class. Systematic sampling was used to select the sample schools [36].

The study excluded 944 students who did not answer questions about their family structure or who had a grandparent family. All 59,096 students who took the survey were included in the study. This was a government-approved statistical survey, and the consent procedure was approved by the institutional review board of the KCDC. The Institutional Review Board at the Catholic University of Korea reviewed and approved the design of this study (IRB approval number: MC19ZESI0089).

### Measures

#### Family structure

Family structure was measured by having the students list family members who were currently living together. The options for the question were “father,” “stepfather,” “mother,” and “stepmother.” We classified the responses into four groups: “Two-parent family (intact family),” “single-mother family,” “single-father family,” and “restructured family.” We defined restructured families as families with stepfathers or stepmothers. The restructured families could have one or two parents.

#### Perceived academic achievement

Perceived academic achievement (PAA) was measured using the question “In the last 12 months, how are your school grades?,” for which answers were recorded using a five-point scale (“high,” “middle high,” “middle,” “middle low,” and “low”). These data were inverse coded and analyzed.

#### Health behavior

For the use or experience of smoking a cigarette, electronic cigarettes or heated tobacco products, drinking a sip of alcohol, drug use, and sexual relations ever in one’s life, there were two possible responses: “Yes” or “No.” Internet use was measured by asking the respondents “In the last 30 days, how many hours of internet use have you had per day on average for purposes other than learning?” Based on a survey of internet overdependence [37], these responses were classified into two groups of “≤ 120 min” and “> 120 min,” referring to the average internet usage time of 120 min per day for adolescents. Obesity was assessed by calculating the body mass index from the weight and height data. Physical activity was assessed using the following question: “In the last seven days, how many days have you had more than 60 minutes of physical activity per day where your heart rate increased from normal?” Responses ranged from “none” to “seven days a week.” Based on the intermediate physical activity criteria of the International Physical Activity Questionnaire, physical activity for at least 3 days a week was classified as participating in vigorous-intensity physical activity [38].

#### Mental health

Depression and suicidal ideation were measured with the questions “Have you experienced sadness or despair to the point that you stopped your daily routine for two weeks?” and “In the past 12 months, have you ever thought of committing suicide?” The response options were “Yes” or “No.” We measured perceived stress and perceived health status with the following questions: “How much stress do you usually feel?” and “How healthy do you usually feel?” Students could respond “very much,” “somewhat,” “average,” “not so much,” or “not at all.” These respondents were categorized into one of two groups: an “average or below-average group” and an “above-average group.” Perceived health status was listed as “very healthy,” “healthy,” “average,” “unhealthy,” or “very unhealthy.” We also categorized respondents into two groups based on health status: “average or above-average” or “below average.”

### Statistical analysis

All statistical analyses were performed using the SPSS 25.0 software (IBM, Armonk, NY, 2017). Based on the analytical guidelines of the KCDC, complex sample weights were applied to reflect nationally representative samples. Descriptive statistics were used to identify sociodemographic and health behavior characteristics according to the family structure of the subjects. A chi-square test was conducted to examine the differences between groups. A complex sample logistic regression was performed to calculate the odds ratios (ORs) and the 95% confidence interval (CI) of health behaviors and mental health by the family structure of the subjects after adjusting for gender, age and perceived family economic status. T-tests and an analysis of variance of the complex sample general linear model were used to examine the association of family structure with the health behaviors, mental health, and academic achievements of the subjects. The significance level was set at *P* < 0.05.

## Results

Table 1 shows the number and general characteristics of students in each family structure group. In total, 55,025 students (93%) were from intact families and 4071 (7%) were from non-intact families. Among non-intact families, single-mother families numbered 1619 (40%), single-father families numbered 1278 (31%), and restructured families numbered 1174 (29%). Two-parent families (13.5%) reported the highest academic achievement, with restructured families at 9.3%. In the low academic achievement group, two-parent families constituted 9.2% and restructured families 20.1%.

**Table 1:** General characteristics and perceived academic achievement of participants in each family structure

Variables	All Participants (*n* = 59,096)	Two-parent family (*n* = 55,025)	Single-mother family (*n* = 1619)	Single-father family (*n* = 1278)	Restructured family (*n* = 1174)	*p-value*
Age (Mean ± SD)	15.1 ± 0.02	15.1 ± 0.02	15.3 ± 0.04	15.4 ± 0.05	15.3 ± 0.08	
Gender						< 0.001
Boy	29,826 (52.1)	27,818 (51.8)	768 (49.5)	689 (57.1)	551 (50.4)	
Girls	29,270 (47.9)	27,207 (48.2)	851 (50.5)	589 (42.9)	623 (49.6)	
School grade						< 0.001
Middle school 1st grade	9692 (14.5)	9089 (14.7)	220 (12.0)	208 (12.8)	175 (12.7)	
Middle school 2nd grade	9949 (15.7)	9344 (15.9)	231 (13.2)	204 (14.6)	170 (13.7)	
Middle school 3rd grade	10,103 (16.3)	9431 (16.3)	276 (15.3)	207 (14.1)	189 (14.7)	
High school 1st grade	9123 (15.9)	8529 (16.0)	264 (17.1)	166 (13.3)	164 (14.6)	
High school 2nd grade	9903 (17.8)	9139 (17.6)	299 (19.3)	218 (18.8)	247 (22.7)	
High school 3rd grade	10,326 (19.9)	9493 (19.5)	329 (23.1)	275 (26.4)	229 (21.7)	
Education level of mother						< 0.001
9 years	770 (1.5)	677 (1.2)	74 (4.3)	–	19 (1.6)	
12 years	16,991 (23.6)	16,066 (29.2)	611 (38.1)	–	314 (27.8)	
14 years or more	29,443 (53.7)	28,639 (53.1)	545 (34.4)	–	259 (23.3)	
I don’t know	10,238 (17.1)	9642 (16.5)	389 (23.1)	–	207 (16.2)	
Education level of father						< 0.001
9 years	927 (1.3)	823 (1.4)	–	74 (5.6)	30 (2.7)	
12 years	14,234 (28.5)	13,518 (24.2)	–	466 (37.7)	250 (21.4)	
14 years or more	31,102 (50.4)	30,505 (57.0)	–	366 (30.2)	231 (21.9)	
I don’t know	10,772 (16.3)	10,178 (17.4)	–	372 (26.6)	222 (18.5)	
Perceived family economic status						< 0.001
High	23,832 (40.8)	22,963 (42.1)	260 (16.2)	304 (24.1)	305 (25.6)	
Middle	27,537 (46.0)	25,672 (46.4)	694 (42.3)	616 (47.5)	555 (47.1)	
Low	7727 (13.2)	6390 (11.5)	665 (41.5)	358 (28.4)	314 (27.3)	
Perceived academic achievement						< 0.001
High	8069 (13.3)	7533 (13.5)	128 (7.5)	94 (7.5)	103 (9.3)	
Middle-High	15,351 (25.4)	14,426 (26.0)	322 (20.3)	264 (21.3)	208 (17.1)	
Middle	17,526 (29.4)	16,247 (29.7)	467 (29.3)	330 (25.5)	291 (24.0)	
Middle-Low	13,249 (22.0)	11,872 (21.6)	463 (28.2)	354 (27.7)	351 (29.5)	
Low	5845 (9.9)	4947 (9.2)	239 (14.8)	236 (18.0)	221 (20.1)	

Weighted percentages following complex sample analysis.

Table 2 presents the frequency of health behavior and mental health status of students by family structure. The use of conventional and electronic cigarettes increased in order of two-parent families (14.1, 7.3%), single-mother families (19.9, 11.4%), single-father families (22.8, 14.1%), and restructured families (27.5, 14.6%). In the heated tobacco product category, two-parent families were the lowest (2.5%) and single-father families the highest (7.2%). The majority of restructured families (60.6%) reported “Yes” in the drinking category, with single-father families at 51%, single-mother families at 47.1%, and two-parent families at 41.5%. Students from single-father families had a significantly greater use of drugs (2.5%) and the internet (50.3%) compared to students from other families. Adolescents who had physical activity less than 3 days a week were most often from single-mother families (71.2%) and least often from single-father families (66.9%). In terms of obesity and sexual experience, 16.3 and 16.7% of restructured family reported in the affirmative, respectively, and for two-parent families it was 14.3 and 5.0%, respectively. In the mental health category, restructured families reported a higher prevalence of depression (34.4%), suicidal ideation (21.4%), above-average perceived stress (49.5%), and below-average perceived health (10.0%) compared to other families; two-parent families had the lowest prevalence (26.6, 12.8%; 40.0%; 6.5%).

**Table 2:** Health behaviors and mental health status of participants by family structure

Variables		Two-parent family (*n* = 55,025)	Single-mother family (*n* = 1619)	Single-father family (*n* = 1278)	Restructured family (*n* = 1174)	*p-value*
Health behavior						
Conventional cigarette use	No	47,689 (85.9)	1301 (80.1)	992 (77.2)	859 (72.5)	< 0.001
	Yes	7336 (14.1)	318 (19.9)	286 (22.8)	315 (27.5)	
Electronic cigarette use	No	51,351 (92.7)	1439 (88.6)	1121 (85.9)	1015 (85.4)	< 0.001
	Yes	3674 (7.3)	180 (11.4)	157 (14.1)	159 (14.6)	
Heated tobacco product use	No	53,762 (97.5)	1561 (96.5)	1205 (92.8)	1105 (93.2)	< 0.001
	Yes	1263 (2.5)	58 (3.5)	73 (7.2)	69 (6.8)	
Drinking	No	32,854 (58.5)	866 (52.9)	652 (49.0)	481 (39.4)	< 0.001
	Yes	22,171 (41.5)	753 (47.1)	626 (51.0)	693 (60.6)	
Drug use	No	54,590 (99.2)	1594 (98.5)	1252 (97.5)	1150 (97.7)	< 0.001
	Yes	435 (0.8)	25 (1.5)	26 (2.5)	24 (2.3)	
Internet use (week day)	≤2 h	24,733 (60.7)	580 (51.4)	419 (49.7)	396 (53.2)	< 0.001
	> 2 h	16,190 (39.3)	537 (48.6)	408 (50.3)	412 (46.8)	
Physical activity (7 days)	≥72 h	18,293 (32.7)	491 (28.8)	418 (33.1)	383 (32.2)	< 0.001
	< 72 h	36,732 (67.3)	1128 (71.2)	860 (66.9)	791 (67.8)	
Obesity	No	47,237 (85.7)	1373 (84.9)	1076 (84.0)	991 (83.7)	0.024
	Yes	7788 (14.3)	246 (15.1)	202 (16.0)	183 (16.3)	
Sexual experience	No	52,461 (95.0)	1512 (93.0)	1145 (88.0)	989 (83.3)	< 0.001
	Yes	2564 (5.0)	107 (7.0)	133 (12.0)	185 (16.7)	
Mental Health						
Depression	No	40,467 (73.4)	1095 (67.7)	882 (69.4)	753 (65.6)	< 0.001
	Yes	14,558 (26.6)	524 (32.3)	396 (30.6)	421 (34.4)	
Suicide ideation	No	48,000 (87.2)	1366 (84.1)	1060 (83.0)	915 (78.6)	< 0.001
	Yes	7025 (12.8)	253 (15.9)	218 (17.0)	259 (21.4)	
Perceived stress	Average or below-average	32,989 (60.0)	892 (55.2)	704 (55.7)	592 (50.5)	< 0.001
	Above average	22,036 (40.0)	727 (44.8)	574 (44.3)	582 (49.5)	
Perceived health	Average or above-average	51,557 (93.5)	1474 (90.6)	1169 (90.8)	1065 (90.0)	< 0.001
	Below average	3468 (6.5)	145 (9.4)	109 (9.2)	109 (10.0)	

Weighted percentages following complex sample analysis.

Table 3 shows ORs that were controlled for gender, age and perceived family economic status of health behavior and mental health according to family structure. In terms of health behaviors, when based on two-parent families, restructured families had the most influential use of conventional cigarettes (2.23), electronic cigarettes (2.05), heated tobacco products (2.51), drinking (2.08), and sexual experience (3.56). The highest OR of internet use was for single-father families (1.31) rather than other families. In the association of mental health with family structure, the ORs of depression, suicidal ideation, above-average perceived stress, and below-average perceived health were 1.33 times, 1.70 times, 1.36 times, and 1.26 times, respectively, greater for restructured families compared with two-parent families. Also, the risk of conventional cigarettes, electronic cigarettes, drinking, sexual experience, increased in the order of two-parent families, single-mother families, single-father families, and restructured families.

**Table 3:** The association of family structure with health behavior and mental health (OR^a^ and 95% CI)

Variables	Two-parent (*n* = 55,025)	Single-mother family (*n* = 1619)	Single-father family (*n* = 1278)	Restructured family (*n* = 1174)
	OR	OR (95% CI)	OR (95% CI)	OR (95% CI)
Health behavior				
Conventional cigarette use	1.00	1.41 (1.23, 1.63)	1.49 (1.27, 1.74)	2.23 (1.92, 2.59)
Electronic cigarette use	1.00	1.58 (1.32, 1.88)	1.67 (1.38, 2.02)	2.05 (1.72, 2.44)
Heated tobacco product use	1.00	1.39 (1.02, 1.90)	2.12 (1.58, 2.85)	2.51 (1.94, 3.25)
Drinking	1.00	1.12 (1.01, 1.25)	1.28 (1.14, 1.45)	2.08 (1.82, 2.38)
Internet use (2 h</week day)	1.00	1.14 (1.00, 1.30)	1.31 (1.11, 1.54)	1.27 (1.08, 1.50)
Physical activity (< 3 days/week)	1.00	1.09 (0.98, 1.21)	0.98 (0.86, 1.12)	0.96 (0.84, 1.10)
Obesity	1.00	0.99 (0.86, 1.15)	1.04 (0.89, 1.21)	1.14 (0.96, 1.35)
Sexual experience	1.00	1.41 (1.10, 1.80)	1.99 (1.62, 2.44)	3.56 (2.99, 4.24)
Mental health				
Depression	1.00	1.16 (1.04, 1.30)	1.12 (0.98, 1.28)	1.33 (1.17, 1.51)
Suicide ideation	1.00	1.08 (0.94, 1.24)	1.24 (1.06, 1.46)	1.70 (1.47, 1.97)
Perceived stress above average	1.00	1.03 (0.92, 1.15)	1.11 (1.01, 1.24)	1.36 (1.20, 1.54)
Perceived health below average	1.00	1.02 (0.86, 1.21)	1.17 (0.94, 1.45)	1.26 (1.01, 1.57)

^a^Adjusted by gender, age and perceived family economic status; *OR* Odds ratios, *CI* Confidence intervals

The associations of PAA with health behavior, mental health, and family structure are shown in Table 4. Smoking including conventional smoking (*β* = − 0.40, *p* = < 0.001), electronic cigarettes (*β* = − 0.44, *p* = < 0.001), and heated tobacco products (*β* = − 0.45, *p* = < 0.001) and drinking (*β* = − 0.23, *p* = < 0.001) and internet use (*β* = − 0.30, *p* = < 0.001) were associated with lower PAA after controlling for gender, age, perceived family economic status and family structure. Similar associations were examined in sexual experience. Poor mental health including depression (*β* = − 0.15, *p* = < 0.001), suicidal ideation (*β* = − 0.15, *p* = < 0.001), perceived stress (*β* = − 0.09, *p* = < 0.001), and perceived health (*β* = − 0.13, *p* = < 0.001) was associated with lower PAA after controlling for gender, age, perceived family economic status and family structure. In family structure, adolescents from single-mother (*β* = − 0.08, *p* = < 0.001), single-father (*β* = − 0.24, p = < 0.001), and restructured families (*β* = − 0.32, *p* = < 0.001) were associated with lower PAA compared to adolescents from two-parent families after controlling for gender, age and perceived family economic status. Also, the effect after additionally controlling for health behavior and mental health was significant for single-mother (*β* = − 0.08, *p* = < 0.001), single-father (*β* = − 0.17, *p* = < 0.001), and restructured families (*β* = − 0.21, *p* = < 0.001).

**Table 4:** The associations of perceived academic achievement with health behavior, mental health and family structure

Variables	Model 1: adjusted for gender, age and perceived family economic status			Model 2: additionally adjusted for family structure			Model 3: additionally adjusted for health behavior and mental health		
	*β*	95% CI	*p*	*β*	95% CI	*p*	*β*	95% CI	*p*
Health behavior									
Conventional cigarette use	−0.40	− 0.43, − 0.37	< 0.001	− 0.40	− 0.43, − 0.38	< 0.001	–	–	–
Electronic cigarette use	−0.43	− 0.47, − 0.39	< 0.001	−0.44	− 0.48, − 0.40	< 0.001	–	–	–
Heated tobacco product use	− 0.42	− 0.49, − 0.36	< 0.001	− 0.45	− 0.52, − 0.38	< 0.001	–	–	–
Drinking	− 0.23	− 0.25, − 0.21	< 0.001	− 0.23	− 0.25, − 0.21	< 0.001	–	–	–
Internet use (2 h</week day)	− 0.30	− 0.33, − 0.28	< 0.001	− 0.30	− 0.32, − 0.28	< 0.001	–	–	–
Sexual experience	− 0.18	− 0.23, − 0.13	< 0.001	− 0.19	− 0.24, − 0.14	< 0.001	–	–	–
Mental Health									
Depression	−0.15	− 0.17, − 0.13	< 0.001	−0.15	− 0.18, − 0.13	< 0.001	–	–	–
Suicide ideation	−0.14	− 0.17, − 0.11	< 0.001	−0.15	− 0.17, − 0.12	< 0.001	–	–	–
Perceived stress above average	−0.09	− 0.11, − 0.07	< 0.001	− 0.09	− 0.11, − 0.07	< 0.001	–	–	–
Perceived health below average	−0.13	− 0.17, − 0.09	< 0.001	−0.13	− 0.17, − 0.95	< 0.001	–	–	–
Family Structure^a^									
Single-mother	−0.08	− 0.13, − 0.02	< 0.001	–	–	–	− 0.08	− 0.14, − 0.01	< 0.001
Single-father	− 0.24	−0.31, − 0.17	< 0.001	–	–	–	− 0.17	−0.26, − 0.08	< 0.001
Restructured family	− 0.32	−0.39, − 0.24	< 0.001	–	–	–	− 0.21	−0.29, − 0.12	< 0.001

^a^Reference group is two-parent group; 95% CI: 95% confidence intervals

## Discussion

This study used nationally representative data on 59,096 South Korean adolescents. We examined the association of diverse family structures with health behavior, mental health, and PAA among adolescents. Adolescents from two-parent families had the highest PAA and those from restructured families had the lowest. Also, in the low-PAA group, the proportion of adolescents increased in the order of two-parent families, single-mother families, single-father families, and restructured families. These findings suggest that family structure could be a risk factor that might have negative effects on adolescents’ academic achievements, especially those from non-intact families. These findings are consistent with previous studies that reported associations between poor academic achievement and non-intact families [31, 32, 34, 39]. Adolescents need parental support for their school-related demands, including academic performance, which is closely related to the family structure [40]. However, single parents may have less time to devote to their children’s school lives than two-parent households due to the many social demands. In fact, children from non-intact families receive less academic encouragement and support on average than those from two-parent families [41, 42].

We found an increasing gradient of OR from two-parent family to restructured families in “smoking,” “using e-cigarettes,” “drinking,” “sexual experience,” after adjusting for gender, age and perceived family economic status. These findings suggest that the level of risk factors affecting the health behavior of adolescents might differ depending on the family structure, and that it is necessary to better understand the association between diverse family structures and health. Our results are consistent with previous studies showing that adolescents in non-intact families are more exposed to the risk of poor health behaviors and mental health [12, 16, 43, 44]. Previous studies reported that good parent-child communication partially reduced smoking, drinking, and drug use [12]. Substance use in adolescents has been found to have a more dangerous association in single-father families than in single-mother families, which can be attributed to the differences between fathers and mothers. Fathers tend to spend less time at home with their children and are more likely to be at work than mothers [2], and mothers are more interested in their children’s daily lives [45] and are more likely to advise them on health issues such as substance use [46]. In terms of parent-child communication, fathers exhibited worse communication than mothers [12]. Previous studies reported that sexual behavior was more related to negative events than to family structure or income. The experience of negative events and sexual behavior in each family structure may need to be further studied later [43, 47]. The poor mental health of adolescents from non-intact families can be understood from various perspectives. Compared to their peers, they have few economic resources [4, 48], lack opportunities to participate [49] in leisure activities, and struggle with the school curriculum, risk of being exposed to bullying; this is higher in single-parent and restructured families than in two-parent ones [6, 44, 50]. Exposure to bullying and violence can affect mental health and lead to behavioral problems [51].

We found that in the categories of smoking, drinking, sexual experience, depression, suicidal ideation, stress, and poor perceived health, reconstructed families had a stronger relationship than the other family structures. Previous studies have reported that restructured families are likely to suffer from interpersonal difficulties such as parental conflicts compared to other families, including single-parent families, and are exposed to domestic violence and abuse [44, 52, 53]. Also, there may be tension among the children of different parents [54]. Elucidating the differences in the physical and mental health of adolescents among these family structures will help to better understand adolescents and establish strategies for health promotion intervention.

In our study, non-intact families were more associated with internet use (more than 2 hours/week) than two-parent families. This result is consistent with previous studies showing that internet addiction is higher in single-parent families than two-parent ones [15]. Our study found that single-father families were more closely associated with internet use than single-mother ones, but a previous study reported that when parents and adolescents had a very bad relationship, the mother factor was more associated with internet addiction than the father factor [15]. Also, negative emotions such as depression, anxiety, and feelings of inferiority that adolescents in non-intact families usually felt were associated with the risk of internet addiction when accompanied by academic stress [55]. Since our study measured “internet use” based on time spent online, our results need to be interpreted carefully when judging addiction. A further study on the relationship between family structure and internet use and addiction is likely needed. The present study showed that the association of family structure with physical activity and obesity was not significant, and this is consistent with a previous meta-analysis study [13] that found no evidence that children of single-parent families need special measures to improve their physical activity levels. Also, in our study, single parents had a lower level of education than two-parent households, and previous studies reported that parents’ low level of education is related to adolescents being overweight [16]. The relationship between family structure and obesity is likely to require further research.

We found that health behaviors and mental health were closely related to PAA even after adjusting for the family structure. A previous study reported that up to 24% of PAA variables were explained in relation to health behavior and academic achievement even after controlling family structure [56]. Our findings were consistent with a previous study that reported that the use of tobacco and alcohol were related to poor academic achievement [57]. According to problem behavior theory, both substance use and poor academic achievement are caused by similar fundamental psychogenic and social risks [58]. The pre- and post-relationship between substance use and poor academic achievement is difficult to fully understand, but most studies agree that substance use and poor academic achievement are in a negative relationship [57, 59]. Our study supports a previous study that reported a link between internet addiction and low academic performance [60]. Also, our study found an association between low PAA and sexual experience. This result is consistent with a previous study that reported that adolescents who engage in sexual intercourse at an early age may undergo a change in attitude, including a decrease in interest in academic activities [61].

In our study, depression, suicidal ideation, high perceived stress, and poor perceived health were associated with low PAA. A previous cohort study reported that academic achievement had a positive correlation with good mental health, hope, life satisfaction, and self-worth [62]. In addition, other previous studies reported that academic performance and poor mental health were negatively related, and that experienced stress was a barrier to academic performance [62, 63]. Our study found a relationship between perceived health and low PAA. In a previous study, the association between perceived health status and academic performance was not significant [63]. This association needs to be further studied. In our study, non-intact families were associated with low PAA compared to adolescents from intact families. Future plans for most non-intact families included the children’s social and educational success and survival. However, those in a socially difficult situation may not be able to plan for their future, resulting in them neglecting their studies and becoming less motivated because they must focus on meeting their current needs [7]. A lack of motivation and awareness was associated with low academic achievement [57].

A clear linear gradient was observed when gender, age and perceived family economic status were adjusted, and linear gradient was maintained when health behaviors and mental health were added. Previous studies reported several reasons for the association of non-intact families with low academic achievement. Adolescents from non-intact families have a lower SES than those from two-parent families, and SES is an important factor in predicting academic achievement. Also, emotional support, encouragement, assistance, and homework support from parents are associated with adolescents’ good academic performance, with adolescents from non-intact families generally having less access to these social resources than those from intact families [34, 64, 65].

The strengths of this study are as follows. First, to the best of our knowledge, this study is meaningful in being the first to make use of nationally representative data on South Korean adolescents to understand their health behaviors, mental health, and PAA by family structure. Second, our study specifically examined the relationship between family structure as a social determinant of health and health behavior, mental health, and PAA, and the findings regarding these relationships are detailed and comprehensive. These results can thus serve as evidence for prioritizing health education for adolescents and will help set the direction of research related to family structure in the future. Our study has limitations that should be considered. First, this study was a cross-sectional one; thus, causal inferences could not be determined. Further longitudinal studies must be conducted to understand the potential ways that family structures may affect the lives of adolescents. Second, because the study utilized secondary data, we were unable to select variables of interest. Among non-intact families, whether parents have custody or not could affect children, but our study did not distinguish custody. Further research is needed that considers detailed classifications in order to understand single parents more deeply. Third, there may have been some self-expression bias because this study was based on self-reporting.

## Conclusion

This study showed that the family structure is a significant factor in adolescent health behavior, mental health, and perceived academic achievement. Adolescents who experience a transition in their family structure may be more vulnerable to health risks and exhibit lower academic achievement compared to those from an intact family. The focus should be on prioritizing interventions that consider the family structure in order to address health inequality in adolescents.

## Data Availability

The datasets for the study are available from the corresponding author on reasonable request. KYRBS data can be accessed and downloaded from the KCDC website (https://www.cdc.go.kr/yhs/).

## Abbreviations

SES: Socioeconomic status
KYRBS: The Korean youth risk behavior web-based survey
KCDC: The Korea centers for disease control and prevention
PAA: Perceived academic achievement
OR: Odds ratio
CI: Confidence interval
